# Supplementary material for: Alterations in the Milk Metabolome of Dairy Cows Supplemented with Different Levels of Calcium Propionate in Early Lactation
Source: Metabolites. 2022 Jul 27;12(8):699. doi: 10.3390/metabo12080699 (PMC9415114; doi:10.3390/metabo12080699)
Supplement: Supplementary file 1 [file metabolites-12-00699-s001.zip › Supplementary materials.pdf]

Alterations in the milk metabolome of dairy cows supplemented with different levels of calcium propionate in early lactation

Fan Zhang [1,2], Yiguang Zhao [1], Hui wang [1], Xuemei Nan [1], Yue Wang [1], Yuming Guo [2,*] and Benhai Xiong [1,*]

[1] State Key Laboratory of Animal Nutrition, Institute of Animal Sciences, Chinese Academy of Agricultural Sciences, Beijing 100193, China

[2] State Key Laboratory of Animal Nutrition, College of Animal Science and Technology, China Agricultural University, Beijing 100193, China

*Corresponding author: Yuming Guo, Email: guoyum@cau.edu.cn; Benhai Xiong, Email: xiongbenhai@caas.cn

Table S1. The metabolites across all milk samples from different feeding levels of calcium propionate in early lactation dairy cows.

Table S2. Identification of significantly differential metabolites from the comparisons of LCaP compared to CON, MCaP compared to CON, HCaP compared to CON, and HCaP compared to MCaP. CON: control group, without calcium propionate addition; LCaP: low calcium propionate, the calcium propionate addition level was 200 g/d per cow; MCaP: medium calcium propionate, the calcium propionate addition level was 350 g/d per cow; HCaP: high calcium propionate, the calcium propionate addition level was 500 g/d per cow.

As **Table S1** and **Table S2** are very large, we have uploaded them in another Excel file.

Table S3. The detailed information about enriched metabolic pathways related to the significantly differential metabolites between LCaP and CON.

Pathway id	Pathway	Total	Hits	Raw p	FDR	Impact	Cpd hits
bta00053	Ascorbate and aldarate metabolism	49	1	0.135	0.271	0.020	cpd:C00879 D-Galactarate;
bta00360	Phenylalanine metabolism	72	1	0.194	0.290	0.014	cpd:C11457 3-(3-Hydroxyphenyl) propanoic acid;
bta00592	alpha-Linolenic acid metabolism	44	1	0.122	0.271	0.023	cpd:C16308 Traumatic Acid;
bta00520	Amino sugar and nucleotide sugar metabolism	108	1	0.278	0.334	0.009	cpd:C00357 N-Acetyl-D-Glucosamine 6-Phosphate;
bta00750	Vitamin B6 metabolism	28	1	0.079	0.271	0.036	cpd:C00847 4-Pyridoxic acid;
bta01100	Metabolic pathways	1712	2	1	1	0.001	cpd:C00847 4-Pyridoxic acid; cpd:C00357 N-Acetyl-D-Glucosamine 6-Phosphate;

Table S4. The detailed information about enriched metabolic pathways related to the significantly differential metabolites between MCaP and CON.

Pathway id	Pathway	Total	Hits	Raw p	FDR	Impact	Cpd hits
bta00290	Valine, leucine and isoleucine biosynthesis	23	1	0.115	0.212	0.043	cpd:C00141 Alpha-ketoisovaleric acid;
bta00280	Valine, leucine and isoleucine degradation	42	1	0.201	0.301	0.024	cpd:C00141 Alpha-ketoisovaleric acid;
bta00410	beta-Alanine metabolism	32	2	0.011	0.135	0.063	cpd:C00986 1,3-Diaminopropane; cpd:C00106 Uracil;
bta00260	Glycine, serine and threonine metabolism	50	1	0.235	0.307	0.020	cpd:C00986 "1,3-Diaminopropane";
bta00230	Purine metabolism	95	2	0.085	0.172	0.021	cpd:C00360 2-Deoxyadenosine 5-monophosphatecpd:C00212 Adenosine;
bta00330	Arginine and proline metabolism	78	1	0.343	0.412	0.013	cpd:C00986 1,3-Diaminopropane;
bta00240	Pyrimidine metabolism	65	2	0.043	0.172	0.031	cpd:C00105 Uridine 5-monophosphate; cpd:C00106 Uracil;
bta04022	cGMP-PKG signaling pathway	10	1	0.051	0.172	0.100	cpd:C00212 Adenosine;
bta04024	cAMP signaling pathway	25	1	0.124	0.213	0.040	cpd:C00212 Adenosine;
bta04071	Sphingolipid signaling pathway	15	1	0.076	0.172	0.067	cpd:C00212 Adenosine;
bta04080	Neuroactive ligand-receptor interaction	52	1	0.243	0.307	0.019	cpd:C00212 Adenosine;
bta00770	Pantothenate and CoA biosynthesis	28	2	0.009	0.135	0.071	cpd:C00141 Alpha-ketoisovaleric acid; cpd:C00106 Uracil;
bta00860	Porphyrin and chlorophyll metabolism	142	1	0.542	0.566	0.007	cpd:C02191 Protoporphyrin IX;

bta04216	Ferroptosis	29	1	0.143	0.229	0.034	cpd:C00418 Mevalonic acid;
bta04270	Vascular smooth muscle contraction	16	1	0.081	0.172	0.063	cpd:C00212 Adenosine;
bta01210	2-Oxocarboxylic acid metabolism	134	1	0.521	0.566	0.007	cpd:C00141 Alpha-ketoisovaleric acid;
bta00900	Terpenoid backbone biosynthesis	45	1	0.214	0.302	0.022	cpd:C00418 Mevalonic acid;
bta01230	Biosynthesis of amino acids	128	1	0.504	0.566	0.008	cpd:C00141 Alpha-ketoisovaleric acid;
bta04924	Renin secretion	17	1	0.086	0.172	0.059	cpd:C00212 Adenosine;
bta04923	Regulation of lipolysis in adipocytes	14	1	0.071	0.172	0.071	cpd:C00212 Adenosine;
bta05032	Morphine addiction	8	1	0.041	0.172	0.125	cpd:C00212 Adenosine;
bta05012	Parkinson disease	15	1	0.076	0.172	0.067	cpd:C00212 Adenosine;
bta05034	Alcoholism	10	1	0.051	0.172	0.100	cpd:C00212 Adenosine;
bta01100	Metabolic pathways	1712	7	1	1	0.004	cpd:C00212 Adenosine; cpd:C00105 Uridine 5-monophosphate; cpd:C02191 Protoporphyrin IX; cpd:C00418 Mevalonic acid; cpd:C00360 2-Deoxyadenosine 5-monophosphate; cpd:C00106 Uracil; cpd:C00141 Alpha-ketoisovaleric acid;

Table S5. The detailed information about enriched metabolic pathways related with the significantly differential metabolites between HCaP and CON.

Pathway id	Pathway	Total	Hits	Raw p	FDR	Impact	Cpd hits
bta00053	Ascorbate and aldarate metabolism	49	1	0.315	0.715	0.020	cpd:C00191 D-Glucuronate;
bta00051	Fructose and mannose metabolism	54	1	0.342	0.715	0.019	cpd:C00392 D-Mannitol;
bta00380	Tryptophan metabolism	81	1	0.469	0.715	0.012	cpd:C01717 Kynurenic acid;
bta00130	Ubiquinone and other terpenoid-quinone biosynthesis	92	1	0.514	0.715	0.011	cpd:C00156 4-Hydroxybenzoate;
bta00040	Pentose and glucuronate interconversions	55	1	0.347	0.715	0.018	cpd:C00191 D-Glucuronate;
bta00360	Phenylalanine metabolism	72	2	0.101	0.639	0.028	cpd:C00156 4-Hydroxybenzoate; cpd:C00805 Salicylic acid;
bta00230	Purine metabolism	95	1	0.525	0.715	0.011	cpd:C00330 Deoxyguanosine;
bta00240	Pyrimidine metabolism	65	2	0.085	0.639	0.031	cpd:C00105 Uridine 5-monophosphate; cpd:C00813 Barbituric acid;
bta00520	Amino sugar and nucleotide sugar metabolism	108	1	0.573	0.715	0.009	cpd:C00191 D-Glucuronate;
bta00562	Inositol phosphate metabolism	47	1	0.305	0.715	0.021	cpd:C00191 D-Glucuronate;
bta00790	Folate biosynthesis	57	1	0.357	0.715	0.018	cpd:C00156 4-Hydroxybenzoate;
bta00860	Porphyrin and chlorophyll metabolism	142	1	0.677	0.715	0.007	cpd:C02191 Protoporphyrin IX;
bta00620	Pyruvate metabolism	31	1	0.212	0.715	0.032	cpd:C01251 Homocitrate;
bta04152	AMPK signaling pathway	22	2	0.011	0.215	0.091	cpd:C00389 Quercetin; cpd:C00757 Berberine;
bta01210	2-Oxocarboxylic acid metabolism	134	1	0.655	0.715	0.007	cpd:C01251 Homocitrate;
bta02010	ABC transporters	126	1	0.631	0.715	0.008	cpd:C00392 D-Mannitol;

bta01230	Biosynthesis of amino acids	128	1	0.637	0.715	0.008	cpd:C01251 Homocitrate;
bta04976	Bile secretion	97	1	0.533	0.715	0.010	cpd:C00805 Salicylic acid;
bta01100	Metabolic pathways	1712	9	1	1	0.005	cpd:C00389 Quercetin; cpd:C01717 Kynurenic acid; cpd:C01251 Homocitrate; cpd:C00805 Salicylic acid; cpd:C00156 4-Hydroxybenzoate; cpd:C00191 D-Glucuronate; cpd:C00105 Uridine 5-monophosphate; cpd:C02191 Protoporphyrin IX; cpd:C00330 Deoxyguanosine;

Table S6. The detailed information about enriched metabolic pathways related to the significantly differential metabolites between HCaP and MCaP.

Pathway id	Pathway	Total	Hits	Raw p	FDR	Impact	Cpd hits
bta00100	Steroid biosynthesis	50	1	0.257	0.355	0.020	cpd:C01724 Lanosterol;
bta00130	Ubiquinone and other terpenoid-quinone biosynthesis	92	1	0.425	0.464	0.011	cpd:C00423 trans-cinnamate;
bta00270	Cysteine and methionine metabolism	61	1	0.305	0.385	0.016	cpd:C03145 N-Formylmethionine;
bta00380	Tryptophan metabolism	81	1	0.385	0.440	0.012	cpd:C01717 Kynurenic acid;
bta00052	Galactose metabolism	46	1	0.239	0.355	0.022	cpd:C00492 Raffinose;
bta00360	Phenylalanine metabolism	72	1	0.350	0.420	0.014	cpd:C00423 trans-cinnamate;
bta00400	Phenylalanine, tyrosine and tryptophan biosynthesis	35	1	0.187	0.299	0.029	cpd:C00296 Quinate;
bta00230	Purine metabolism	95	2	0.103	0.205	0.021	cpd:C00212 Adenosine; cpd:C00144 Guanosine 5-monophosphate;
bta01523	Antifolate resistance	17	1	0.095	0.205	0.059	cpd:C00144 Guanosine 5-monophosphate;
bta04022	cGMP-PKG signaling pathway	10	2	0.001	0.032	0.200	cpd:C00144 Guanosine 5-monophosphate; cpd:C00212 Adenosine;
bta04024	cAMP signaling pathway	25	1	0.137	0.253	0.040	cpd:C00212 Adenosine;
bta04071	Sphingolipid signaling pathway	15	1	0.084	0.205	0.067	cpd:C00212 Adenosine;
bta04080	Neuroactive ligand-receptor interaction	52	1	0.266	0.355	0.019	cpd:C00212 Adenosine;
bta04270	Vascular smooth muscle contraction	16	1	0.090	0.205	0.063	cpd:C00212 Adenosine;
bta04742	Taste transduction	32	1	0.172	0.296	0.031	cpd:C00144 Guanosine 5-monophosphate;
bta04740	Olfactory transduction	8	1	0.046	0.205	0.125	cpd:C00144 Guanosine 5-monophosphate;
bta04744	Phototransduction	8	1	0.046	0.205	0.125	cpd:C00144 Guanosine 5-monophosphate;
bta04923	Regulation of lipolysis in adipocytes	14	1	0.079	0.205	0.071	cpd:C00212 Adenosine;
bta04924	Renin secretion	17	1	0.095	0.205	0.059	cpd:C00212 Adenosine;
bta02010	ABC transporters	126	1	0.535	0.559	0.008	cpd:C00492 Raffinose;

bta05012	Parkinson disease	15	1	0.084	0.205	0.067	cpd:C00212 Adenosine;
bta05032	Morphine addiction	8	1	0.046	0.205	0.125	cpd:C00212 Adenosine;
bta05034	Alcoholism	10	1	0.057	0.205	0.100	cpd:C00212 Adenosine;
bta01100	Metabolic pathways	1712	5	1	1	0.003	cpd:C01717 Kynurenic acid; cpd:C00423 trans-cinnamate; cpd:C00144 Guanosine 5-monophosphate; cpd:C00212 Adenosine; cpd:C01724 Lanosterol;

a

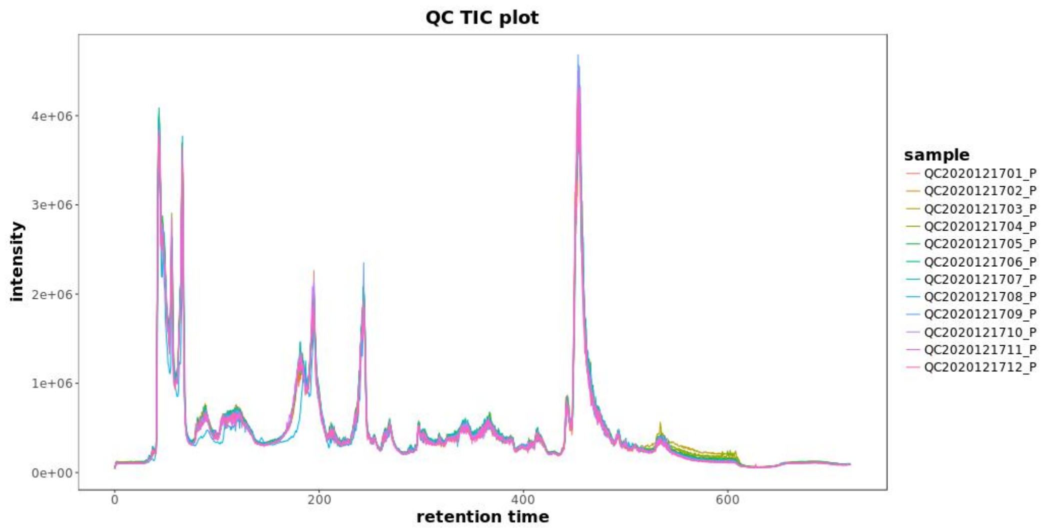

b

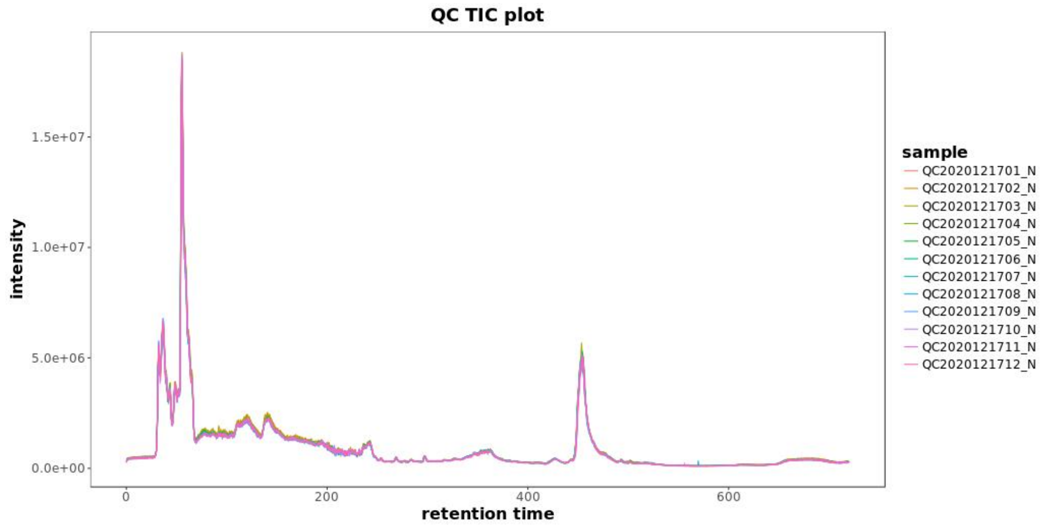

Supplemental Figure S1. Total ion chromatogram of quality control (QC) samples analyzed in the positive (a) and negative (b) ion modes.

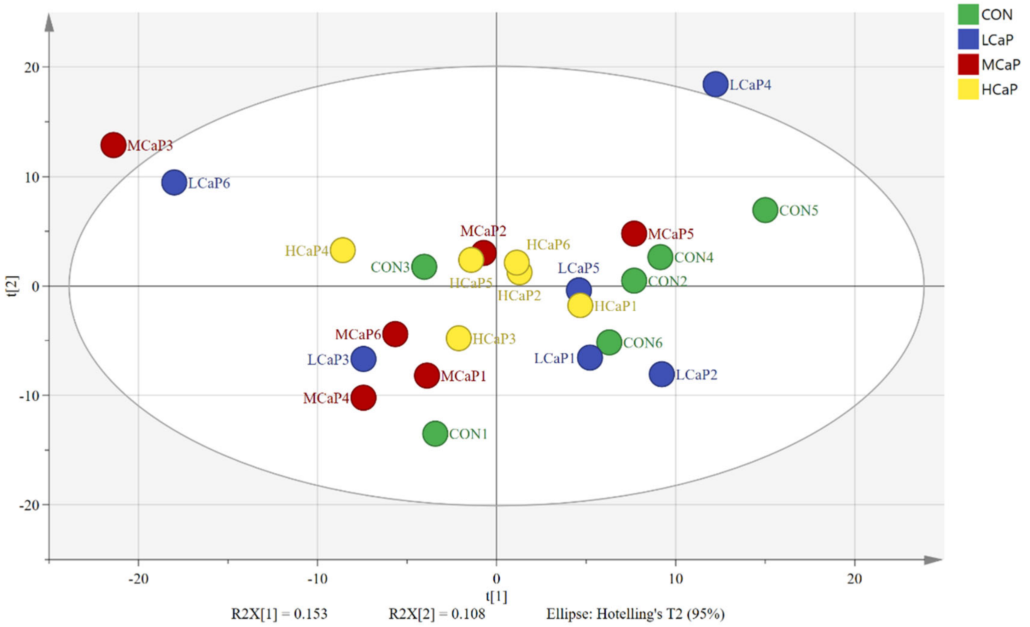

Supplemental Figure S2. Principal component analysis (PCA) score plot for the milk samples collected from different feeding levels of calcium propionate in early lactation dairy cows. CON: control group, without calcium propionate addition; LCaP: low calcium propionate, the calcium propionate addition level was 200 g/d per cow; MCaP: medium calcium propionate, the calcium propionate addition level was 350 g/d per cow; HCaP: high calcium propionate, the calcium propionate addition level was 500 g/d per cow.

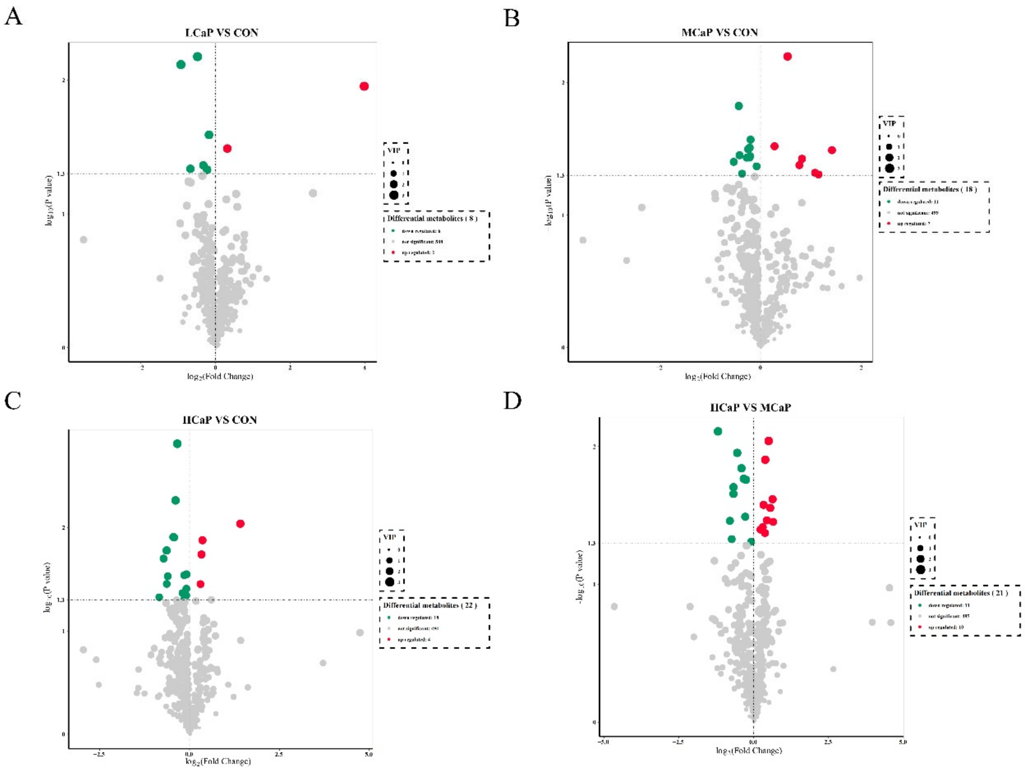

Supplemental Figure S3. Volcano plots for the comparisons of LCaP compared to CON (A), MCaP compared to CON (B), HCaP compared to CON (C), and MCaP compared to HCaP (D) from the milk metabolite profiles of the experimental cows. The x-axis is the $1og_2$ (FC) value, and the y-axis represents $-log_{10}$ (*P* value). The dot size indicates the variable importance in the projection (VIP) value. CON: control group, without calcium propionate addition; LCaP: low calcium propionate, the calcium propionate addition level was 200 g/d per cow; MCaP: medium calcium propionate, the calcium propionate addition level was 350 g/d per cow; HCaP: high calcium propionate, the calcium propionate addition level was 500 g/d per cow.
